# Reemergence of Cosmopolitan Genotype Dengue Virus Serotype 2, Southern Vietnam

**DOI:** 10.3201/eid2910.230529

**Published:** 2023-10

**Authors:** Vi T. Tran, Rhys P.D. Inward, Bernardo Gutierrez, Nguyet M. Nguyen, Phong T. Nguyen, Isabelle Rajendiran, Tam T. Cao, Kien T.H. Duong, Moritz U.G. Kraemer, Sophie Yacoub

**Affiliations:** Oxford University Clinical Research Unit, Ho Chi Minh City, Vietnam (V.T. Tran, N.M. Nguyen, K.T.H. Duong, S. Yacoub);; University of Oxford, Oxford, UK (R.P.D. Inward, B. Gutierrez, M.U.G. Kraemer, S. Yacoub);; Universidad San Francisco de Quito, Quito, Ecuador (B. Gutierrez);; Hospital for Tropical Diseases, Ho Chi Minh City (P.T. Nguyen, T.T. Cao);; Imperial College London, UK (I. Rajendiran).

**Keywords:** dengue virus, viruses, arboviruses, mosquito-borne infections, DENV serotype 2, climatic factors, Vietnam, vector-borne infections

## Abstract

We performed phylogenetic analysis on dengue virus serotype 2 Cosmopolitan genotype in Ho Chi Minh City, Vietnam. We document virus emergence, probable routes of introduction, and timeline of events. Our findings highlight the need for continuous, systematic genomic surveillance to manage outbreaks and forecast future epidemics.

Dengue virus (DENV) represents a major public health concern globally and in Vietnam, where an estimated 1.6 million cases occur each year (*1*). Clinical manifestations range from fever to severe organ dysfunction (*2*). DENV includes 4 distinct serotypes (DENV 1–4), which have evolved into distinguishable genotypes; all are transmitted primarily by *Aedes aegypti* mosquitoes (*3*). DENV is endemic in both urban and peri-urban areas of Vietnam with substantial seasonal temporal and spatial variation. Although all 4 DENV serotypes have circulated in Vietnam, DENV-1 and DENV-2 have been most prevalent over the past 20 years. 

In 2022, Ho Chi Minh City, Vietnam, experienced a 3-fold increase in reported DENV cases compared with 2020 and 5-fold compared with 2021 (*4*,*5*). Possible drivers of transmission include climate factors because optimal temperatures and humidity increase vector abundance (*6*), reduced population immunity because of lower rates of transmission in previous years (*6*), and possible introduction of new serotypes or genotypes or diversification of circulating lineages (*7*). Scarcity of available data on DENV circulation by lineage in Vietnam limits testing those hypotheses. Our study explored the DENV lineages circulating in Ho Chi Minh City over the past 5 years, aiming to provide a detailed assessment of DENV dynamics in Vietnam. 

We randomly selected 362 samples from dengue patients enrolled in research studies (reviewed by the ethics committee and approved by the internal review board of the hospital) at the Hospital for Tropical Diseases in Ho Chi Minh City during 2017–2022. Of those patients, 303 (83.7%) tested positive for dengue using quantitative reverse transcription PCR. DENV-2 was predominant (72.3%); DENV-1 accounted for 23.1% and DENV-4 for 4.6% of positive samples. From those samples, we sequenced 45 DENV-2 viral envelope (E) genes. We amplified E genes using PCR, and after PCR product purification, we conducted Sanger sequencing on samples with nucleic acid concentrations >10 ng/μL. We supplemented the new sequences with a background dataset of DENV-2 genome and E-gene sequences from southern and southeast Asia (Appendix 1). 

We constructed maximum-likelihood trees for all DENV-2 genotypes, as well as a time-scaled tree in which we estimated ancestral node locations and conducted root-to-tip regression analyses. Our results showed DENV-2 replaced DENV-1 as the predominant serotype in 2019, and multiple DENV-2 genotypes were cocirculating (Appendix 1 Figure 1). Seventeen sequences belonged to Asia I genotype, which has been established in the region since 2006; another 28 sequences were identified as sporadically detected Cosmopolitan genotype. Cosmopolitan genotype virus strain has displayed signs of reemergence, with indicators of >3 distinct recent introductions of clades A, B, and C into southern Vietnam (Appendix 1 Figure 2). Cosmopolitan phylogeny (Figure), constructed similarly to the broader DENV-2 tree, shows most (27/28) sequences from this region clustered within these 3 clades; clades A and B share ancestry with sequences from Indonesia and clade C shares ancestry with sequences from Cambodia. 

**Figure:**
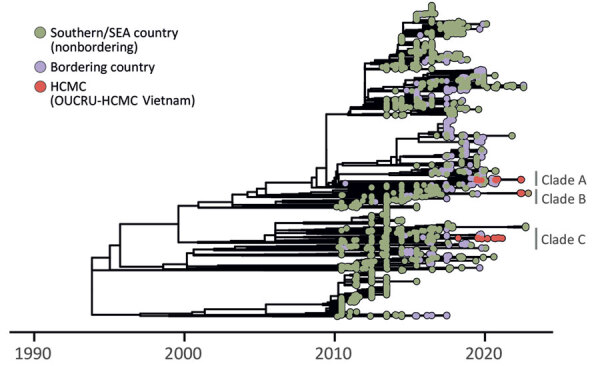
Time-scaled maximum-likelihood phylogenic tree showing emergence of the Cosmopolitan genotype of dengue virus serotype 2 in Vietnam. Clades A–C refer to transmission lineages. HCMC, Ho Chi Minh City; OUCRU, Oxford University Clinical Research Unit; SEA, southeast Asia

The clades were detected across several provinces in southern Vietnam, including all 3 clades in Ho Chi Minh City. Clade A circulated in provinces north and east of Ho Chi Minh City; clades B and C were found in provinces north and west of the city (Appendix 1 Figure 3). Estimated time to the most recent common ancestor suggests that clade A has been in circulation the longest, followed by clades B and C (Appendix 1 Figure 4). These findings indicate continuous introductions of the Cosmopolitan genotype into southern Vietnam over multiple years. Clades A and C have persisted locally for at least half a decade, with approximately 2 years between the earliest most recent common ancestor and the earliest sampling date, suggesting undetected Cosmopolitan genotype might have been cryptically transmitted during multiple dengue seasons. 

Dynamics of DENV prevalence and spread within Vietnam might have been influenced by successive seedings of the Cosmopolitan genotype, thereby increasing likelihood of establishment and sustained transmission (*8*,*9*). Although the Cosmopolitan genotype has circulated in neighboring countries, likely since the late 1990s (Appendix 1 Figures 5, 6), it had not been reported in Vietnam until recently. Mechanisms underlying a specific genotype reemerging after new introductions and subsequent establishment warrant further investigation. Large-scale dengue surveillance in hyperendemic settings like Vietnam is a formidable challenge because it relies on syndromic surveillance and awareness of heterogeneous epidemiologic trends, and limited resources are available to support viral genomic sequencing to identify circulating strains. To refine our understanding of transmission pathways for specific lineages of DENV, improving surveillance using strategies that reduce sampling bias is critical (*10*). We emphasize the importance of continuous, systematic virus sequencing in urban centers in Vietnam and across southeast Asia to swiftly identify novel viral lineages. These strategies, paired with clinical and socioeconomic data, will support preventive measures and outbreak forecasting. 

Appendix 1 Additional information on reemergence of Cosmopolitan genotype of dengue virus serotype 2, southern Vietnam.

Appendix 2 Additional list of acknowledgments.
