# Supplementary material for: Reemergence of Cosmopolitan Genotype Dengue Virus Serotype 2, Southern Vietnam
Source: Emerg Infect Dis. 2023 Oct;29(10):2180–2. doi: 10.3201/eid2910.230529 (PMC10521597; doi:10.3201/eid2910.230529)
Supplement: Appendix 1 — Additional information on reemergence of Cosmopolitan genotype of dengue virus serotype 2, southern Vietnam. [file 23-0529-Techapp-s1.pdf]

Article DOI: https://doi.org/10.3201/eid2910.230529
EID cannot ensure accessibility for Appendix materials supplied by authors. Readers who have difficulty accessing Appendix content should contact the authors for assistance.

Reemergence of Cosmopolitan Genotype Dengue Virus Serotype 2, Southern Vietnam

Appendix 1

E-gene Sequencing

We produced 45 DENV-2 viral envelope (E) gene sequences from human plasma samples, randomly collected from 362 laboratory-confirmed dengue patients, who participated in studies at the Hospital for Tropical Diseases in HCMC, Vietnam, between 2017 and 2022 (*1–4*). All of the studies are approved by Ethics Committees both in Vietnam, at the Hospital for Tropical Disease and also in the UK, at the Oxford Tropical Research Ethics Committee (OxTREC). Of these, 303 (83.7%) were positive with qRT-PCR assay for dengue, DENV-2 was predominant (72.3%), whereas DENV-1 and DENV-4 respectively accounted for 23.1% and 4.6% of positive samples. No samples were positive with DENV-3. Amongst the 219 DENV-2–positive samples, we randomly selected 45 samples with high or medium viremia levels that gave clear bands on agarose gel electrophoresis (median Cp-value for the samples was 25.53 [IQR=23.38–27.29]). The patients' median age was 26 (IQR=17.5–33.5) years. Viral RNA from each sample was isolated using MagNA Pure 96 DNA and Viral NA Small Volume Kit (Roche, 06543588001) in the MagNA Pure 96 System.

DENV RNA was subjected to cDNA synthesis using SuperScript IV First-Strand Synthesis System (Cat # 18091200, Thermo Fisher Scientific) on a Mastercycler system (Eppendorf, Germany) according to the manufacturer's instructions. 13 µL of Mix1 containing 5µL of viral RNA, 1µL of random hexamer primer, 1µL of a mixture of 10mM deoxyribonucleotide triphosphates(dNTPs) and 6 µL of water. A mixture of 7µL of Mix 2 containing 4µL of 5x Reverse Transcript buffer, 1µL of each RNAse inhibitor and Superscript IV

Reverse Transcriptase was added to Mix 1 and made up a final volume of 20μL. cDNA was synthesised by the thermocycler program in Appendix 1 Table 1.

PCR reactions to amplify the E gene were performed as 3-tube reactions using a high-fidelity DNA polymerase (Cat # M0491L, New England Biolabs). The PCR mix contained 1μL dNTPs (0.4mM), 2μL of eluted primers containing both forward and reverse primers (10μM), 3μL of cDNA, and 0.5μL of DNA polymerase (2000 units/ml) diluted to a final volume of 25μL per reaction. The amplification reactions were performed according to the manufacturer's instructions (Appendix 1 Table 2). PCR amplification products were visualized by agarose gel electrophoresis. The primer sets are available in Appendix 1 Table 3.

We purified PCR products using magnetic beads (Cat # A63882, Beckman Courter) and quantified nucleic acid concentration using a NanoDrop 2000/2000c Spectrophotometer (Cat # ND-2000). Sanger sequencing of purified PCR products was carried out for samples with a nucleic acid concentration greater than 10 ng/μL. Sequencing of the complete E gene was achieved by 3 overlapping amplicons. The sequencing reactions were performed with the BigDye Terminator v3.1 Cycle Sequencing Kit (Cat # 4337455) according to the manufacturer's instructions (Appendix 1 Table 4). The reaction was terminated by adding 2μL of 3M sodium acetate and 100mM EDTA. A DNA precipitation step was then carried out with absolute ethanol (97%) and washed with ethanol 70%, and samples were then resuspended in 10μL of Hi-Di formamide. Sanger sequencing was performed using an Applied Biosystems 3130/3130xl Genetic Analyzer. Individual E gene consensus sequences were generated using the CLC Genomic Workbench version 9.0.

Data collation, sequence alignment, and tree building

We collected 6013 of both DENV-2 whole genome (WG) and E gene sequences along with their associated metadata from southern and southeast Asia between 2010-01-01 and 2022-12-19 from GenBank (data downloaded 2022-12-19) (*5*). We removed any sequences with missing metadata, such as the absence of collection date or location, resulting in a total of 5836 sequences (which include 139 sequences from Vietnam). For sequences with incomplete metadata, i.e., yyyy or yyyy-mm, we took the mid-point of that year (e.g., 2011 → 2011-06-15). To facilitate the visualisation of phylogenies, we classified the sequence collection location

information into 4 discrete categories: OUCRU-HCMC Vietnam, Other Vietnam, Border countries that share a border with Vietnam (China, Laos, and Cambodia) and other south and southeast Asia countries that do not share a border with Vietnam.

Due to errors in the serotype labelling within the GenBank metadata, we used Arbovirus typing tool from Genome Detective to identify the serotype and genotype of each sequence (*6*). All entries that were not DENV-2 were removed. We aligned sequences to the DENV-2 reference sequence (NC_001474.2) using MAFFTV.7 (*7*). All sequences were trimmed from position 937 to 2421 to ensure only the E gene was included. Additionally, sequences were further excluded if they had >5% missing or ambiguous sites, leaving a dataset of n = 4497. Lastly, we created a second dataset (n = 2812) containing sequences designated Cosmopolitan by Genome Detective.

After the initial data processing, the cleaned sequence alignment files were used to construct a maximum-likelihood (ML) tree for all DENV-2 genotypes and a separate phylogeny for the Cosmopolitan genotype alone. ML trees were inferred using IQTREE2 (*8*) with a nonparametric ultrafast bootstrap to estimate node support and a Shimoda-Hasegawa approximate likelihood ratio test (SH-aLRT) (*9*) to estimate branch support (command line: Iqtree2 -s -bb 1000 -alrt 1000). We used a TIM2 nucleotide substitution model with empirical base frequencies and a free rate model for rate heterogeneity with four rate categories based on a hierarchical model selection procedure as implemented in the ModelFinder application (*10*). The output ML trees were assessed for temporal signal by selecting the root position that maximizes R^2 of the regression of collection times on genetic distances to the tree root using TempEst v1.5.3 (*11*), removing outliers and reestimating trees when necessary. An outlier was defined as any tip whose genetic distance exceeds four standard deviations of the sample mean for each year. This resulted in a final dataset of 4,496 sequences for all DENV-2 and 2,809 sequences for the Cosmopolitan genotype. The ML tree for the Cosmopolitan genotype was then time-calibrated, informed by tip sampling dates (yyyy-mm-dd), using TreeTime (*12*).

The mugration model (https://github.com/rhysinward/Phylodynamics-HCMC/blob/main/results/confidence.csv) for ancestral node reconstruction implemented within TreeTime (*13*) was used to infer the location of the ancestral node state of the Cosmopolitan genotype along the phylogeny, using the country of origin of each sequence as a discrete trait. To

infer the ancestral state, TreeTime employs a maximum-likelihood framework that estimates the probability of the ancestral states at each node of the phylogeny, given the observed states of its descendants. The most likely location of each node in the phylogeny is then presented as the inferred ancestral location.

Root-to-tip regression plots for the Cosmopolitan ML tree (before time calibration) are available (Appendix 1 Figure 5). All tree visualizations were created using the ggtree package (*13*) within the programming language R.

References

1. Goncalves DDS, Hue KDT, Thuy VT, Tuyet NV, Thi GN, Thi Thuy VH, et al. Assessing the vertical transmission potential of dengue virus in field-reared *Aedes aegypti* using patient-derived blood meals in Ho Chi Minh City, Vietnam. Parasit Vectors. 2020;13:468. PubMed https://doi.org/10.1186/s13071-020-04334-5

2. Tuan Vu T, Clapham H, Huynh VTT, Vo Thi L, Le Thi D, Vu NT, et al. Blockade of dengue virus transmission from viremic blood to *Aedes aegypti* mosquitoes using human monoclonal antibodies. PLoS Negl Trop Dis. 2019;13:e0007142. PubMed https://doi.org/10.1371/journal.pntd.0007142

3. Nguyen NM, Chanh HQ, Tam DTH, Vuong NL, Chau NTX, Chau NVV, et al. Metformin as adjunctive therapy for dengue in overweight and obese patients: a protocol for an open-label clinical trial (MeDO). Wellcome Open Res. 2021;5:160. PubMed https://doi.org/10.12688/wellcomeopenres.16053.2

4. McBride A, Vuong NL, Van Hao N, Huy NQ, Chanh HQ, Chau NTX, et al. A modified Sequential Organ Failure Assessment score for dengue: development, evaluation and proposal for use in clinical trials. BMC Infect Dis. 2022;22:722. PubMed https://doi.org/10.1186/s12879-022-07705-8

5. Clark K, Karsch-Mizrachi I, Lipman DJ, Ostell J, Sayers EW. GenBank. Nucleic Acids Res. 2016;44(D1):D67–72. PubMed https://doi.org/10.1093/nar/gkv1276

6. Vilsker M, Moosa Y, Nooij S, Fonseca V, Ghysens Y, Dumon K, et al. Genome Detective: an automated system for virus identification from high-throughput sequencing data. Bioinformatics. 2019;35:871–3. PubMed https://doi.org/10.1093/bioinformatics/bty695

7. Katoh K, Misawa K, Kuma K, Miyata T. MAFFT: a novel method for rapid multiple sequence alignment based on fast Fourier transform. Nucleic Acids Res. 2002;30:3059–66. PubMed https://doi.org/10.1093/nar/gkf436

8. Minh BQ, Schmidt HA, Chernomor O, Schrempf D, Woodhams MD, von Haeseler A, et al. IQ-TREE 2: New Models and Efficient Methods for Phylogenetic Inference in the Genomic Era. Mol Biol Evol. 2020;37:1530–4. PubMed https://doi.org/10.1093/molbev/msaa015

9. Shimodaira H, Hasegawa M. Multiple Comparisons of Log-Likelihoods with Applications to Phylogenetic Inference. Mol Biol Evol. 1999;16:1114–6. https://doi.org/10.1093/oxfordjournals.molbev.a026201

10. Kalyaanamoorthy S, Minh BQ, Wong TKF, von Haeseler A, Jermiin LS. ModelFinder: fast model selection for accurate phylogenetic estimates. Nat Methods. 2017;14:587–9. PubMed https://doi.org/10.1038/nmeth.4285

11. Rambaut A, Lam TT, Max Carvalho L, Pybus OG. Exploring the temporal structure of heterochronous sequences using TempEst (formerly Path-O-Gen). Virus Evol. 2016;2:vew007–007. PubMed https://doi.org/10.1093/ve/vew007

12. Sagulenko P, Puller V, Neher RA. TreeTime: Maximum-likelihood phylodynamic analysis. Virus Evol. 2018;4:vex042. PubMed https://doi.org/10.1093/ve/vex042

13. Yu G, Smith DK, Zhu H, Guan Y, Lam TTY. ggtree: an r package for visualization and annotation of phylogenetic trees with their covariates and other associated data. Methods Ecol Evol. 2017;8:28–36. https://doi.org/10.1111/2041-210X.12628

Appendix 1 Table 1. Thermocycler program for cDNA synthesis using SuperScript IV First-Strand Synthesis System (Cat # 18091200, Thermo Fisher Scientific)

Mix	Step	Temperature, °C	Time, min
1			
	Unfold RNA	70	7
	poly-T primer binding	Ice	2
2			
	Annealing	50	45
	Elongation	55	15
	Enzyme deactivation	80	10
	Storage	4	Hold

Appendix 1 Table 2. Thermocycler program for PCR DENV with high fidelity DNA polymerase (Cat # M0491L, New England Biolabs)

Step	Temperature (°C)	Time	Cycles
Initial denaturation	98	30 secs	1
Denaturation	98	10 secs	x 40
Annealing	60	1.5 min	
Extension	72	30 secs	
Final extension	72	10 mins	1
Storage	4	Hold	

Appendix 1 Table 3. Primer sets for PCR and sequencing

Name	Forward Primer Sequence (5'–3')	Reverse Primer Sequence (5'–3')
DV2-F1-R1	AGAAGAGAAAAAAGATCAGTGGC	CCATGTTTTCCTGTGTCATTWCC
DV2-F2-R2	GCATTGTGACCTGTGCTATGT	CGAATGGAGGTTCTGCTTCTAT
DV2-F3-R3	AAATAGCAGAAACACAACATGGAAC	TCTGGTGTTATTTGTYTCCACAT

Appendix 1 Table 4. Thermocycler program for Sequencing using the BigDye Terminator v3.1 Cycle Sequencing Kit (Cat # 4337455)

Step	Temperature (ºC)	Time	Cycles
Initial denaturation	96	1 min	1
Denaturation	96	10 secs	x 35
Annealing	50	10 secs	
Extension	60	1 min	
Storage	4	Hold	

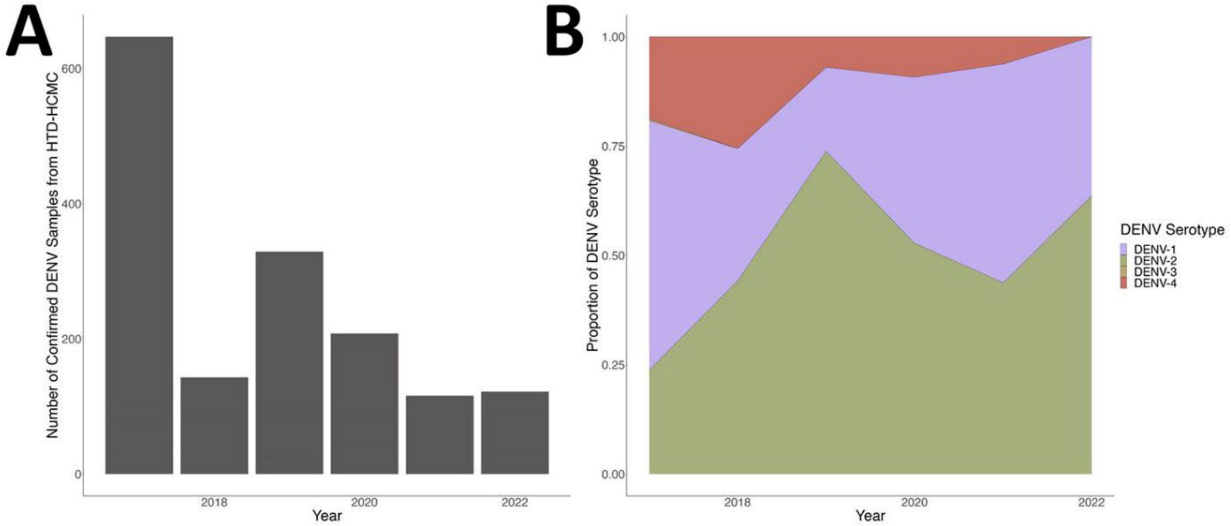

Appendix 1 Figure 1. A) Times series of confirmed cases by NS1 enrolled within studies conducted by Oxford University Clinical Research Unit – Ho Chi Minh City (OUCRU-HCMC) and B) proportion of case-patients participating in studies conducted at OURCU-HCMC assigned to each serotype in HCMC.

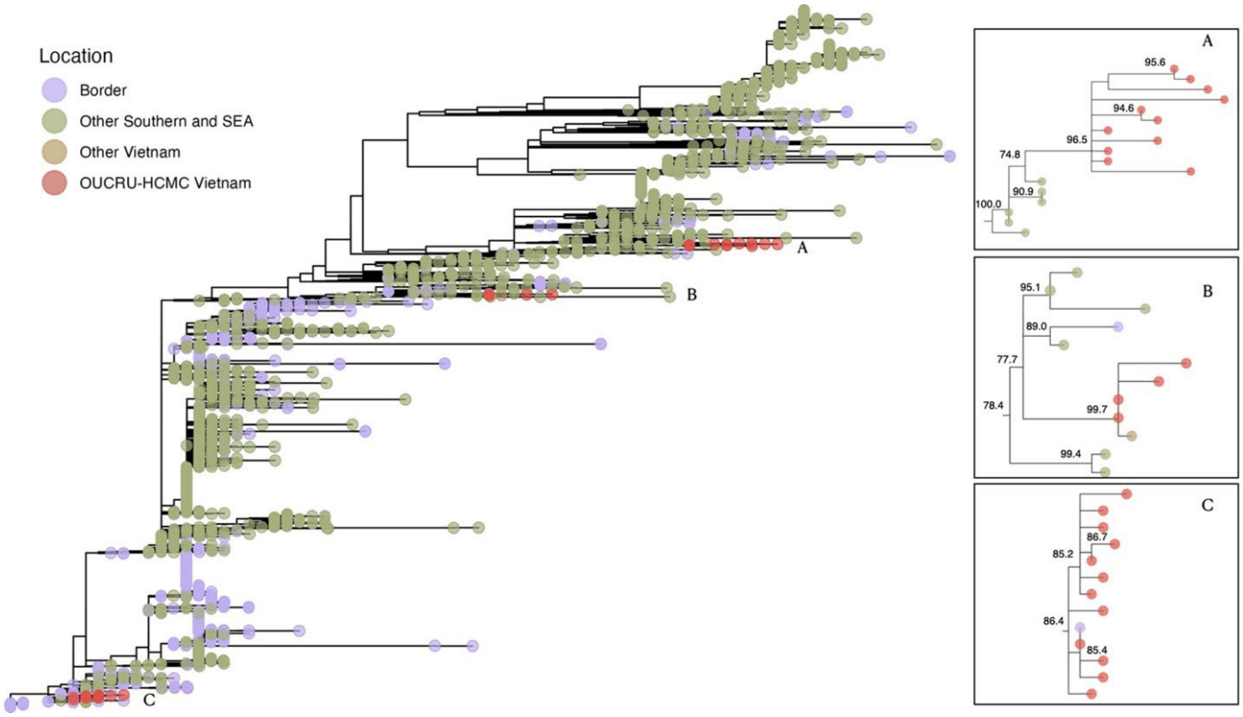

Appendix 1 Figure 2. ML tree showing the Cosmopolitan genotype of DENV-2 within southern and southeast Asia (including Vietnam). Boxes A–C show each transmission lineage circulating within Vietnam alongside their node support values.

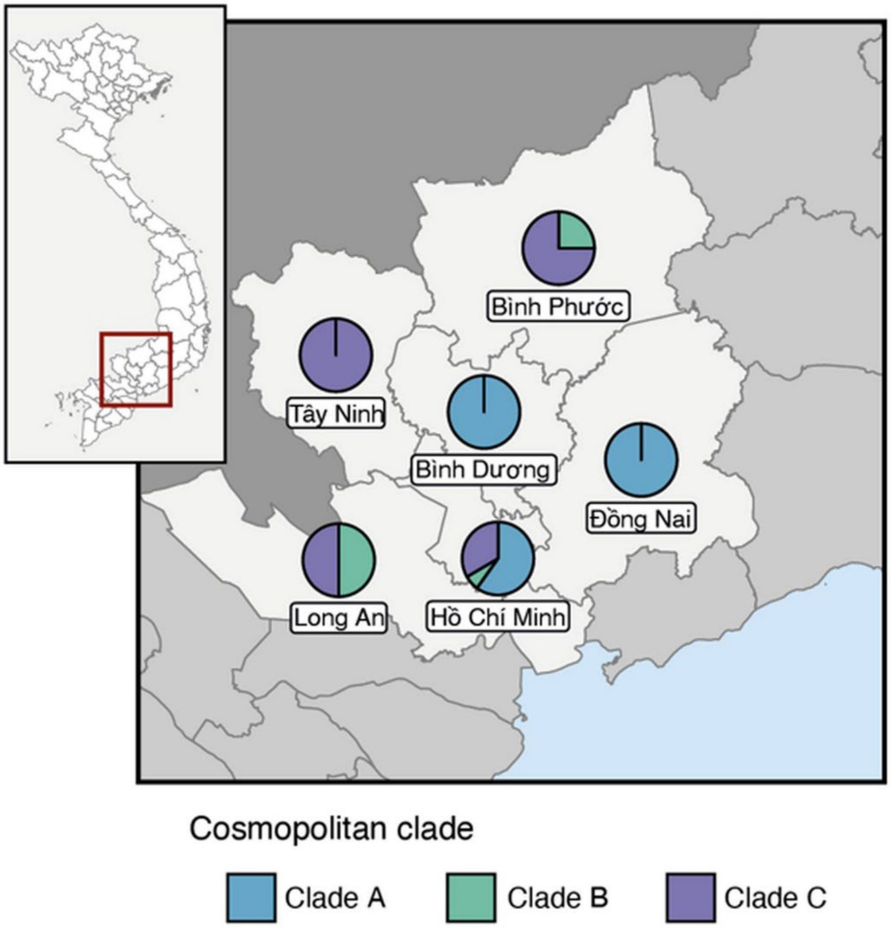

Appendix 1 Figure 3. Spatial distribution of DENV-2 Cosmopolitan clades in southern Vietnam. Clades A, B, and C correspond to independent introductions into Vietnam from other countries and circulated among 6 provinces in southern Vietnam (including Ho Chi Minh City).

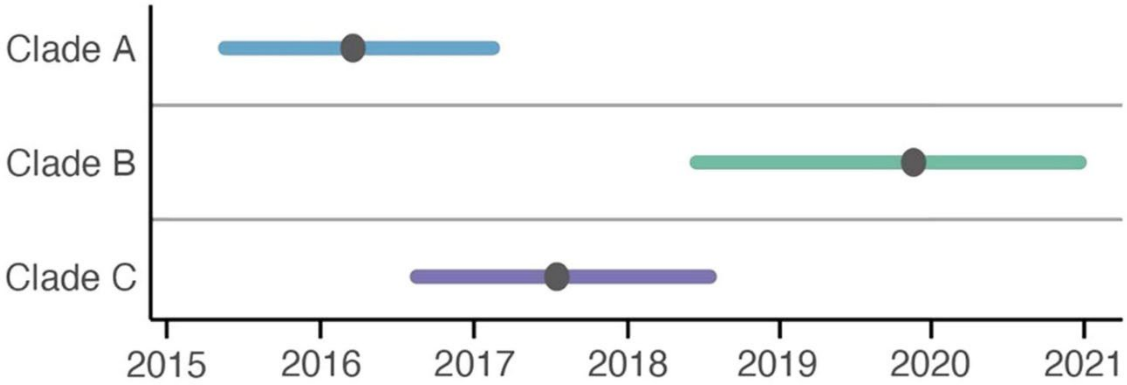

Appendix 1 Figure 4. The inferred TMRCA (black dot) of the first transmission event by each lineage (Clade A, B, C) and their 90% CI (shaded area) by the Cosmopolitan genotype within Vietnam.

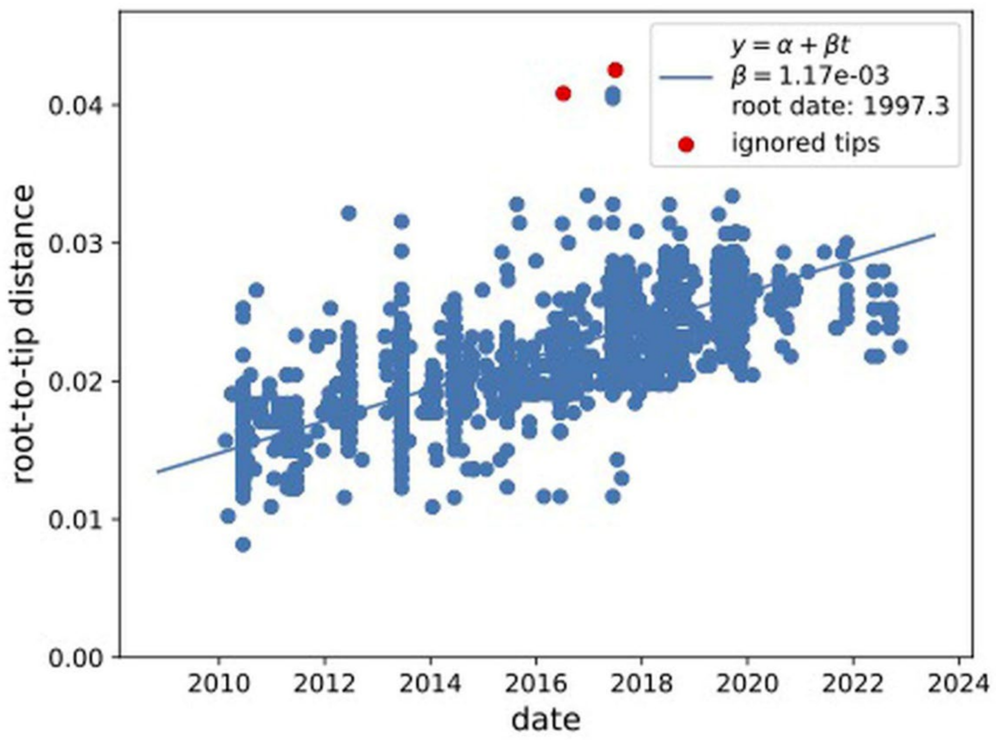

Appendix 1 Figure 5. Root-to-tip regression by TreeTime of the Cosmopolitan DENV-2 dataset.

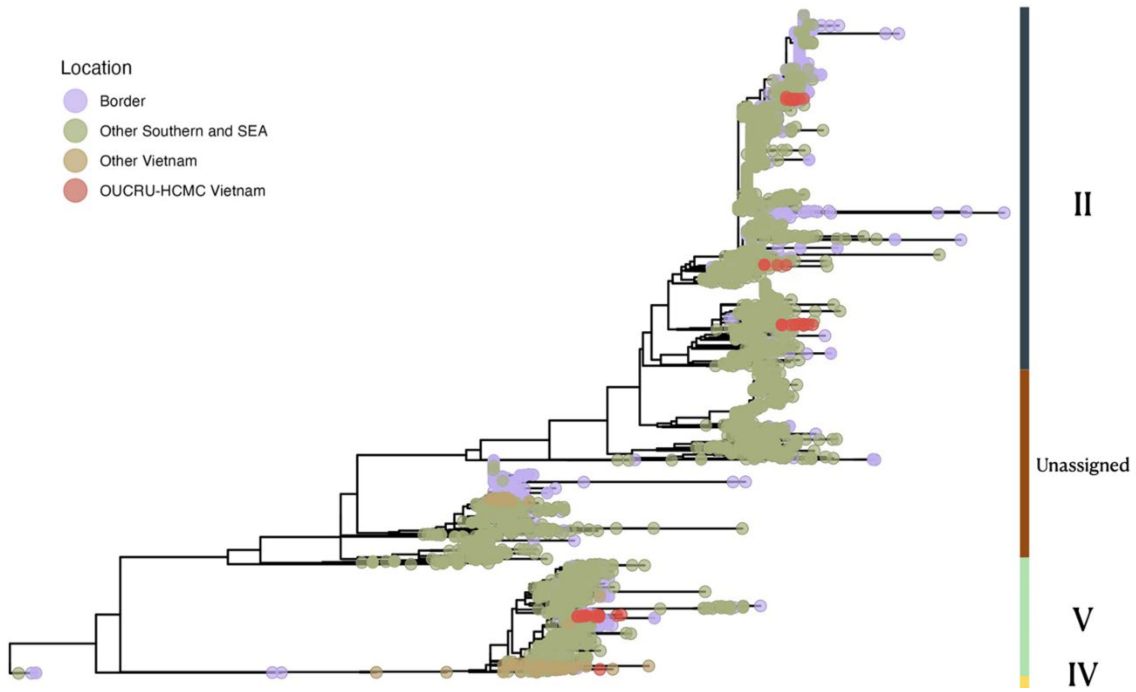

Appendix 1 Figure 6. ML tree showing the genotypes of DENV-2 present within southern and southeast Asia (including Vietnam), 2010–2022.
